# A Persisting Nontropical Focus of Burkholderia pseudomallei with Limited Genome Evolution over Five Decades

**DOI:** 10.1128/mSystems.00726-20

**Published:** 2020-11-10

**Authors:** Jessica R. Webb, Nicky Buller, Audrey Rachlin, Clayton Golledge, Derek S. Sarovich, Erin P. Price, Mark Mayo, Bart J. Currie

**Affiliations:** a Global and Tropical Health Division, Menzies School of Health Research, Charles Darwin University, Darwin, Northern Territory, Australia; b Department of Infectious Diseases, Royal Darwin Hospital, Darwin, Northern Territory, Australia; c Northern Territory Medical Program, Royal Darwin Hospital, Darwin, Northern Territory, Australia; d Department of Primary Industries and Regional Development, Perth, Western Australia, Australia; e Infections West Hollywood Medical Centre, Perth, Western Australia, Australia; f GeneCology Research Centre, University of the Sunshine Coast, Sippy Downs, Queensland, Australia; g Sunshine Coast Health Institute, Sunshine Coast University Hospital, Birtinya, Queensland, Australia; University of Southampton

**Keywords:** *Burkholderia pseudomallei*, clonality, melioidosis, phylogenetics, temperate climate

## Abstract

Burkholderia pseudomallei is the causative agent of the high-mortality disease melioidosis. Although melioidosis is classified as a tropical disease, rare autochthonous cases have been reported from temperate climatic regions, with uncertainty as to whether B. pseudomallei is persistent in the local environment and whether specific genetic mechanisms facilitate the survival of B. pseudomallei outside the tropics. Sporadic cases of melioidosis occurred in a valley region (latitude 31.6°S) in southwest Western Australia, Australia, between 1966 and 1992. We report a new melioidosis cluster in the same region following high rainfall in January 2017. More than 20 animals died, and B. pseudomallei was isolated from four alpacas, a parrot, and three environmental samples taken from the farm where the alpacas resided. Epidemiological data and genomics revealed that two locations on the farm were the probable sources of the alpaca infections. We determined that B. pseudomallei isolates from the 2017 cluster belonged to sequence type 284 (ST-284), as did all isolates recovered from 1966 to 1992. Genomic analysis confirmed that the ST-284 isolates were clonal and contained conserved genomic islands and limited evidence of recombination. We identified protein-coding regions unique to these isolates that might influence the persistence of B. pseudomallei in this temperate region. We demonstrate the environmental persistence of B. pseudomallei in a temperate region for over 50 years, with limited genetic changes suggesting a latent state and with activation, potential aerosolization, and local dispersal following unusually high rainfall.

**IMPORTANCE** Burkholderia pseudomallei is predominantly a tropical pathogen uncommonly found in the environment of temperate climatic regions. It is unclear if introduction into temperate regions is sporadic and temporary or if B. pseudomallei can persist in such environments. B. pseudomallei was identified in the environment of southwest Western Australia with melioidosis cases between 1966 and 1991. We report a new cluster with 23 animal fatalities in the same region from 2017, with B. pseudomallei again being recovered from the environment. Comparison of the isolates from the first and second clusters using genomics revealed a single sequence type, high clonality, and limited recombination, even though the time of recovery of the isolates spanned 51 years. This is a major contrast to the extensive genomic diversity seen in the tropics. Our data support the suggestion that B. pseudomallei has the ability to persist in nontropical environments, potentially in a latent state, and has the ability to activate following favorable conditions (rainfall) and then infect animals and humans.

## Abstract (precis)

Burkholderia pseudomallei is predominantly a tropical pathogen uncommonly found in the environment of temperate climatic regions. It is unclear if introduction into temperate regions is sporadic and temporary or if B. pseudomallei can persist in such environments. B. pseudomallei was identified in the environment of southwest Western Australia with melioidosis cases between 1966 and 1991. We report a new cluster with 23 animal fatalities in the same region from 2017, with B. pseudomallei again being recovered from the environment. Comparison of the isolates from the first and second clusters using genomics revealed a single sequence type, high clonality, and limited recombination, even though the time of recovery of the isolates spanned 51 years. This is a major contrast to the extensive genomic diversity seen in the tropics. Our data support the suggestion that B. pseudomallei has the ability to persist in nontropical environments, potentially in a latent state, and has the ability to activate following favorable conditions (rainfall) and then infect animals and humans.

## INTRODUCTION

Melioidosis is an often fatal disease of humans and animals caused by the Gram-negative sapronotic bacterium Burkholderia pseudomallei. Inoculation, inhalation, and ingestion are the routes of B. pseudomallei infection, with almost all cases being acquired from an environmental source and with the mortality rate in humans being 10 to 40% (1, 2). B. pseudomallei has a comparatively large and complex genome, facilitating survival in diverse hosts and harsh environments (3). This genome has two chromosomes of ∼4 megabase pairs and ∼3 megabase pairs. The smaller chromosome encodes a higher number of accessory functions associated with the adaptation and survival of B. pseudomallei in different ecological niches (3). The B. pseudomallei genome is also subject to high rates of horizontal gene transfer of DNA, which has been essential to its evolution. This has also been important in the development of genomic islands (GIs) variably present throughout the genome, having potential implications in the pathogenesis and survival of B. pseudomallei (3).

B. pseudomallei is predominantly recognized as a tropical pathogen; however, its global distribution has now been extended to include additional tropical and subtropical regions (4–11). The presence of B. pseudomallei in the environment of Northern Territory, Australia, and northeast Thailand is well recognized, with the highest incidence rates of melioidosis being reported from these two regions (12, 13). In Australia, melioidosis has been documented in both the tropics and the temperate regions and was first documented in sheep in 1949 (14) and then in a human in 1950 (15); both of these cases were in tropical north Queensland. On rare occasions, cases of melioidosis have been reported in temperate regions of Australia, typically following severe weather events (10), including the Avon Valley region and surrounds of southwest Western Australia (latitude 31.6°S). Cases of melioidosis were first described in farm animals from this region in 1966, with further animal cases being reported up until 1987, isolation of B. pseudomallei being reported from soil in 1980, and a single human case being reported in a hobby farmer in 1992 (16–19). These southwest Western Australia cases which occurred between 1966 and 1992 have been referred to as the “quarter-century melioidosis outbreak event” (10).

The climate in southwest Western Australia is temperate, with rainfall from June to August and an environment previously predicted to be unfavorable for the persistence of B. pseudomallei (8). Despite this, in February 2017 a new melioidosis cluster was reported from the same region. The second melioidosis infection cluster included 23 alpacas (Vicugna pacos) located at a farm in the region known as Moondyne Farm and a scarlet macaw parrot (Ara macao) located elsewhere in the region, with no human cases being reported. All alpacas died, prompting soil sampling at the property to support the epidemiological investigation. The primary goal of this study was to determine if B. pseudomallei was in the environment where the alpacas died in 2017 and to determine if the genotypes of the isolates were consistent among the animal and environmental sources and matched those of the isolates in the initial outbreak cluster that occurred between 1966 and 1992. An additional aim was to assess the genomic diversity and phylogeography of all strains from the region over the 51 years and to determine if strains from temperate Australia contain protein-coding regions that are absent from tropical Australian strains.

## RESULTS

### Higher-than-average rainfall was detected in southwest Western Australia in January 2017.

**FIG 1:**
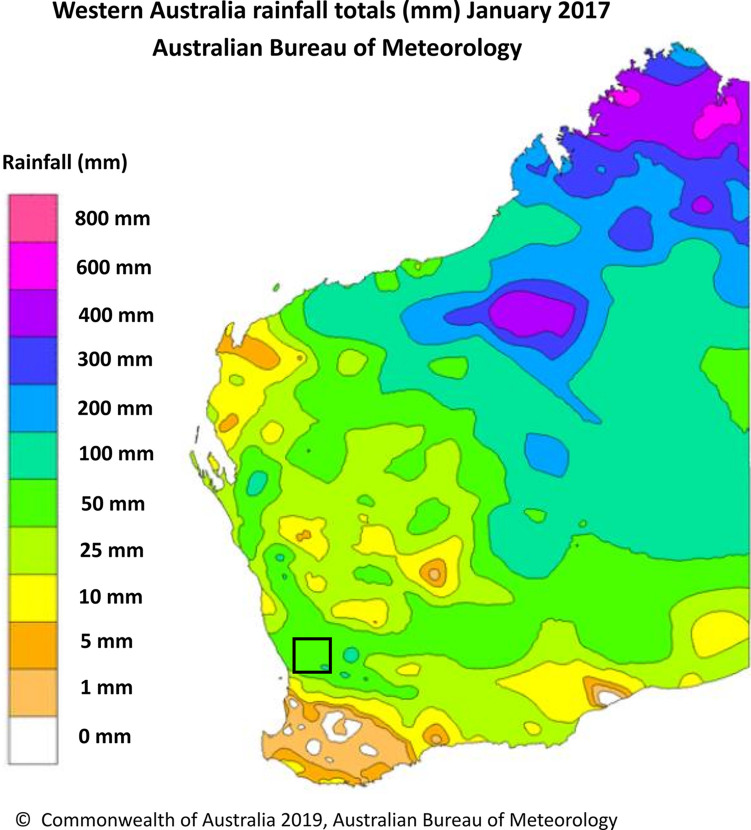
Western Australian rainfall totals (in millimeters) in January 2017, the month prior to the melioidosis outbreak in Moondyne of the Avon Valley region, Western Australia. Moondyne Farm is located within the black square, and the color gradient indicates the amount of rainfall (in millimeters). The image is from the Australian Bureau of Meteorology (www.bom.gov.au).

From 30 years of climatology data from the Australian Bureau of Meteorology, the average rainfall in southwest Western Australia during January is 10 mm. During January 2017, southwest Western Australia received an average reading of 50 to 200 mm of rainfall (Fig. 1), with all rain falling between 29 and 31 January, 17 days prior to the reported alpaca deaths on Moondyne Farm. Rainfall was based on data from three weather stations in southwest Western Australia: Northam, Western Australia, reported 130 mm; Gingin, Western Australia, reported 115 mm; and Pearce, Western Australia, reported 84 mm.

### B. pseudomallei was cultured from animal samples and the alpaca environment.

**FIG 2:**
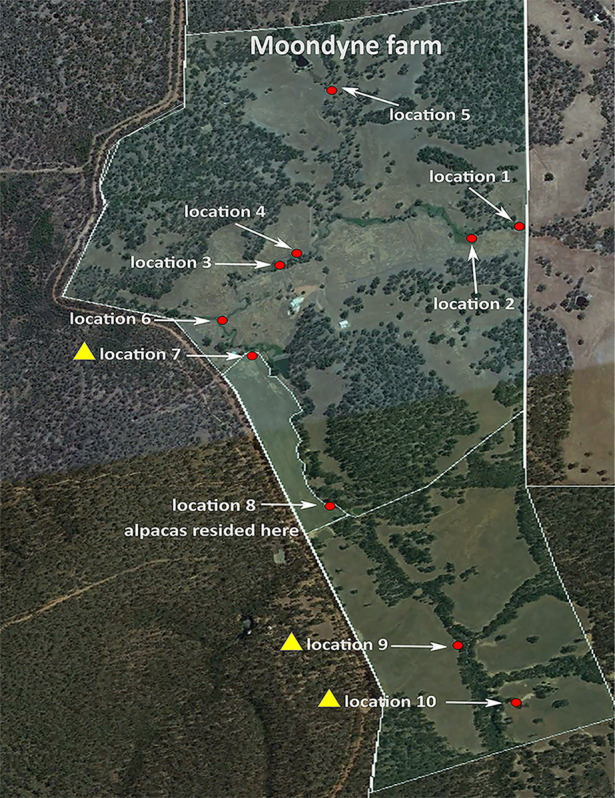
Map of Moondyne Farm (the area within the white border) showing the 10 locations that were environmentally sampled. Locations 7, 9, and 10 were positive for B. pseudomallei (indicated by the yellow triangles), and B. pseudomallei was not identified elsewhere. The image was created using Inkscape 0.92.3.

**FIG 3:**
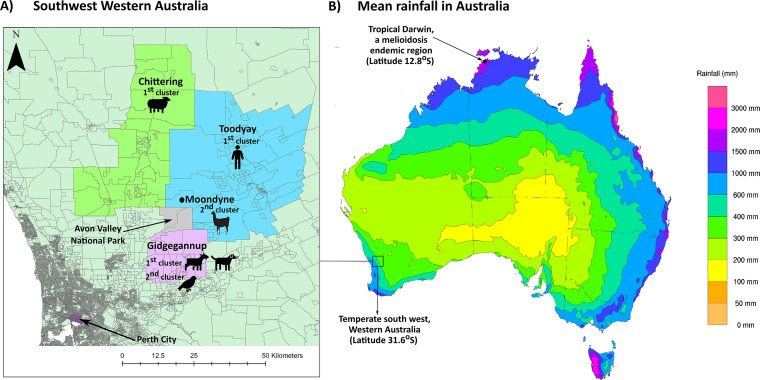
Geography of the region of southwest Western Australia, where the endemicity of melioidosis is low. (A) Geographical origin of B. pseudomallei isolates within southwest Western Australia. Isolates from the first melioidosis cluster are from Chittering and Toodyay, which are in the Avon Valley region of southwest Western Australia, and Gidgegannup, which, despite bordering Avon Valley, is not considered a part of Avon Valley. The isolates from the second melioidosis cluster are from Moondyne, which is located within the Avon Valley or Gidgegannup. The exact locations of the farms are not presented here to maintain privacy. The regions have been segregated and assigned as outlined by the Australian Bureau of Statistics (https://www.abs.gov.au/; statistical area level 3), and the map was created using the ArcGIS program (https://www.arcgis.com/index.html). (B) A map of the mean rainfall in Australia (January 1981 to December 2010) that demonstrates the location of temperate southwest Western Australia in comparison to that of tropical Darwin, where melioidosis is highly endemic. The image and data were supplied by the Australian Bureau of Meteorology (www.bom.gov.au) and adapted with its permission.

Table 1 describes the six animal and four soil B. pseudomallei isolates recovered and the tissue cultured. Figure 2 shows the three locations on the farm with positive soil samples, and Fig. 3 shows the geographical locations of Moondyne and Gidgegannup in southwest Western Australia in comparison to the locations of previous melioidosis cases in that region. The Moondyne Farm owner reported that the alpacas were located at location 8 on the farm and that soil from this site was culture negative, but the adjacent sampling locations were those that were culture positive (Fig. 2).

### All animal and environmental isolates belong to ST-284.

*In silico* multilocus sequence typing (MLST) showed that all nine Moondyne Farm-Avon Valley animal outbreak and soil isolates and the parrot isolate (Table 1, second cluster) belonged to a single sequence type (ST), ST-284. ST-284 was the ST of the human isolates and all animal and environmental isolates recovered from this region between 1966 and 1992 (Table 1, first cluster). ST-284 has not been identified outside of the Avon Valley and surrounding region, being unique to this temperate location among 1,778 STs currently in the global MLST data set. Interrogation of the B. pseudomallei MLST website demonstrated that there were no single-locus variants (SLVs) or double-locus variants (DLVs) of ST-284. Triple-locus variants (TLVs) of ST-284 include ST-320 (Northern Territory, Australia), ST-434 (Northern Territory, Australia), ST-600 (Townsville, Australia), ST-630 (Queensland, Australia), and ST-634 (Queensland, Australia).

**TABLE 1:** ST-284 isolates from the first and second clusters, spanning a 50-year time period

B. pseudomallei isolate	Date collected	ST	Host	Source	Tissue sample	Southwest Western Australia location	Reference or source
First cluster (1966–1992)							
MSHR0157	Nov 1992	284	Human	Clinical	Mediastinal tissue	Toodyay	10
MSHR0160	Jan 1966	284	Sheep	Animal	Spine	Chittering Farm 1	10
MSHR0161	Jan 1966	284	Sheep	Animal	Lung	Chittering Farm 2	10
MSHR0162	Jan 1968	284	Sheep		Abscess	Chittering Farm 1	10
MSHR0163	Jan 1978	284	Sheep	Animal	Lung	Chittering Farm 1	10
MSHR0164	Jan 1978	284	Guinea pig	Animal	Lung	NA^*a*^	10
MSHR0167	Jan 1980	284	Sheep	Animal	Lung	Chittering/Gidgegannup^*b*^	10
MSHR0169	Jan 1980	284	Soil	Environmental	NA	Chittering Farm 1	10
MSHR0170	Jan 1980	284	Soil	Environmental	NA	Chittering Farm 1	10
MSHR0171	Jan 1983	284	Goat	Animal	Lung	Gidgegannup Farm 1	10
MSHR0172	Jan 1985	284	Dog	Animal	Abscess	Gidgegannup Farm 2	10
MSHR0173	Jan 1987	284	Goat	Animal	Lung	Gidgegannup Farm 2	10
Second cluster (2017)							
MSHR10305	May 2017	284	Alpaca A	Animal	Lung	Moondyne Farm location 8^*c*^	This study
MSHR10306	May 2017	284	Alpaca B	Animal	Lung	Moondyne Farm location 8^*c*^	This study
MSHR10307	May 2017	284	Alpaca C	Animal	Lung	Moondyne Farm location 8^*c*^	This study
MSHR10308	May 2017	284	Alpaca C	Animal	Vitreous humor	Moondyne Farm location 8^*c*^	This study
MSHR10309	May 2017	284	Alpaca D	Animal	Lung	Moondyne Farm location 8^*c*^	This study
MSHR10310	May 2017	284	Scarlet macaw parrot	Animal	Tissue sample	Gidgegannup	This study
MSHR10319	May 2017	284	Soil	Environmental	NA	Moondyne Farm location 7^*c*^	This study
MSHR10320	May 2017	284	Soil	Environmental	NA	Moondyne Farm location 9^*c*^	This study
MSHR10380	May 2017	284	Soil	Environmental	NA	Moondyne Farm location 9^*c*^	This study
MSHR10489	May 2017	284	Soil	Environmental	NA	Moondyne Farm location 10^*c*^	This study

a NA, not applicable.

b The sheep from which isolate MSHR0167 was retrieved had been moved several times, and they had spent 6 weeks on Gidgegannup Farm 2 (10).

c See Fig. 2 for the location from which samples were retrieved at Moondyne Farm.

### Phylogenetic analysis of the ST-284 strains from the first and second clusters.

**FIG 4:**
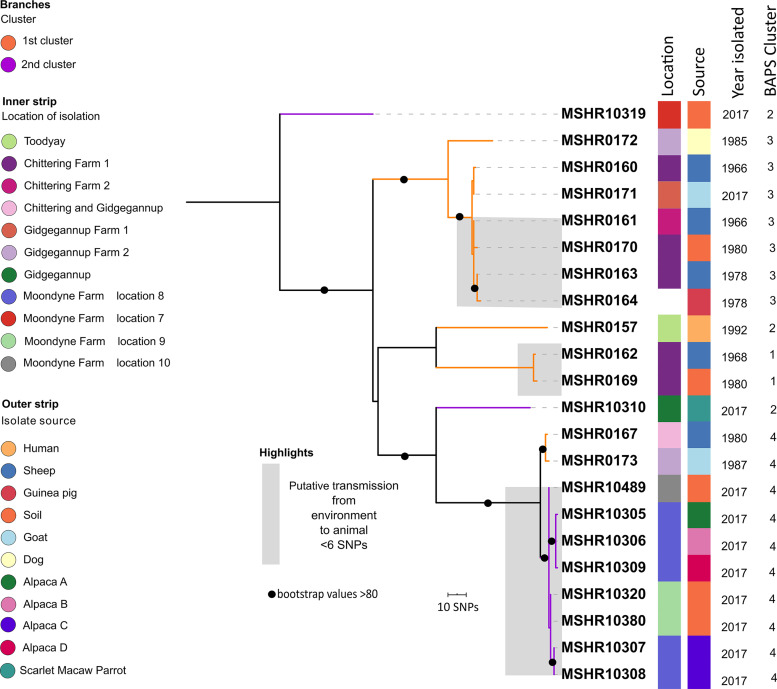
Midpoint-rooted maximum parsimony phylogeny of B. pseudomallei ST-284 isolates (*n* = 22) from southwest Western Australia, based on 532 core-genome SNPs. Bootstrapping revealed that the phylogenetic tree had moderately supported to well-supported nodes, with the branch support values ranging from 80 to 100. Branch colors indicate cluster 1 or cluster 2 (with the first cluster occurring between 1966 and 1992 and the second cluster occurring in 2017); the inner strip delineates the location of isolation; the outer strip delineates the isolate source, which is followed by the year in which the sample was collected and the BAPS cluster for each isolate, as defined by the RhierBAPS program; and the gray highlighted regions delineate transmission events. The geographical location for isolate MSHR0164 is unknown, and so the location of MSHR0164 was not included. Black circles on the branches denote bootstrap values of >80. The consistency index for the tree was 0.9963, and the homoplasy index was 0.0037.

Phylogenetic analysis was initially performed solely on the genomes of the ST-284 isolates from the first and second clusters (*n* = 22) to determine the phylogenetic relatedness of the ST-284 isolates. The 22 genomes were mapped with 60× to 120× coverage against the genome of the reference ST-284 isolate, isolate MSHR0169. Despite the isolation of these B. pseudomallei genomes over more than 5 decades, phylogenetic analysis of the 22 genomes revealed that the 22 ST-284 isolates were clonal, with 532 core-genome single-nucleotide polymorphism (SNP) differences being detected across the ST-284 phylogeny (Fig. 4). The population structure of the 22 isolates was further defined using the RhierBAPS program, based on a core-genome SNP mapping alignment, which divided the 22 isolates into 4 primary sequence clusters (Bayesian analysis of population structure [BAPS] hierarchical level 1). These were further subdivided into 8 lineages (BAPS level 2) (see Table S1 in the supplemental material). Two of the primary sequences that clustered at level 1, sequence clusters 2 and 4, comprised sequences from isolates from both melioidosis time clusters (Table 2), while sequence clusters 1 and 3 consisted of isolates collected during the first southwest Western Australian melioidosis cluster (Table 2). Time cluster 2 included three isolates that were distributed across different branches of the maximum parsimony (MP) tree. The characteristics of each cluster are listed in Table 2. We also constructed a recombination-adjusted phylogeny (Fig. S2); strain clustering was consistent with the phylogenetic clustering observed with the MP SNP tree.

**TABLE 2:** Characteristics of clusters that make up the phylogenetic tree presented in Fig. 4^*a*^

Sequence cluster	No. of isolates		Total no. of SNPs	Longest time difference (yr)	Isolate geographical location(s)	Largest geographical difference (km)
	Cluster 1	Cluster 2				
1	2	0	3	12	Chittering	0
2	1	2	NA^*b*^	25	Gidgegannup, Toodyay, and Moondyne	∼40
3	7	0	49	51	Chittering and Gidgegannup	∼65
4	2	8	19	37	Chittering, Gidgegannup and Moondyne	∼60

a Clustering was determined using the RhierBAPS program and represents level 1 clusters.

b NA, not applicable (consists of isolates that were distributed across different branches of the MP tree [Fig. 4]).

10.1128/mSystems.00726-20.3 TABLE S1 All genomes used in this study. Download Table S1, XLSX file, 0.03 MB. Copyright © 2020 Webb et al. 2020 Webb et al. This content is distributed under the terms of the Creative Commons Attribution 4.0 International license.

The ST-284 isolates did not completely group together on the basis of the year or the location of isolation but were highly conserved (Fig. 4). Eight strains recovered from Moondyne Farm in 2017 grouped together, belonged to level 1 sequence cluster 4, and were conserved with nine SNPs, and they also clustered most closely to isolates (separated by eight SNPs) from two distinct geographical regions that had been isolated 37 years earlier (in 1980 and 1987). One isolate recovered from a parrot in 2017 was distant to the cluster containing the eight Moondyne Farm 2017 isolates and two isolates recovered in the 1980s, being separated by 116 SNPs. The remaining soil isolate (isolate MSHR1039) from Moondyne Farm formed its own lineage that was distinct from the other Moondyne Farm isolates, which were located 146 SNPs away. Level 1 sequence cluster 3 contained a cluster of seven clonal isolates from four geographical regions outside of the Moondyne Farm that were isolated between 1966 and 1985.

For the cluster that occurred in 2017, there were phylogenetic links between the animals and the soil taken from Moondyne Farm (Fig. 4, purple branches). The isolates from alpacas A (isolate MSHR10305), B (isolate MSHR10306), and D (isolate MSHR10309) all clustered together within level 1 sequence cluster 4 and were closely related to the soil isolate (isolate MSHR10489) from location 10 of Moondyne Farm, being separated from the soil sample isolate by only four SNPs. Identical isolates from alpaca C (isolates MSHR10307 and MSHR10308) clustered together in level 1 sequence cluster 4 and were most closely related to soil isolates (isolates MSHR10320 and MSHR10380) from Moondyne Farm location 9, being separated by only three SNPs. The isolate from the scarlet macaw parrot (isolate MSHR10310) clustered separately from the isolates from the alpacas and did not cluster closely to any of the soil isolates from Moondyne Farm, with the closest soil isolate (MSHR10489) being 121 SNPs different from the parrot isolate.

The phylogenetic relatedness of the animal and environmental ST-284 isolates from the first southwest Western Australia cluster (Fig. 4, orange branches) has been described previously (10). Phylogenetic reconstruction of ST-284 strains from the first cluster with the isolates from the second cluster did not considerably alter the phylogenetic structure of the ST-284 cluster 1 isolates (10).

### Southwest Western Australia isolates form a single clade on the global B. pseudomallei phylogeny.

**FIG 5:**
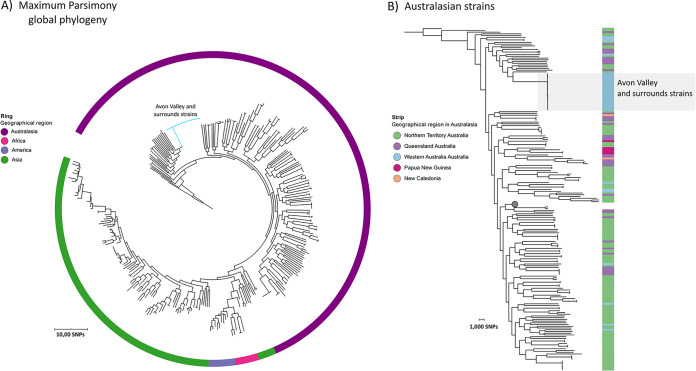
Maximum parsimony phylogenetic analysis of ST-284 isolates on a global scale (*n* = 318). The phylogeny is based on 216,486 core-genome SNPs. The analysis is rooted with MSHR0668, the most ancestral B. pseudomallei strain, as identified in a large phylogenetic study (59). (A) Maximum parsimony phylogeny of global B. pseudomallei genomes, including the 22 southwest Western Australia strains (blue branches). The ring delineates the global geographical region of origin. (B) Positions of the 22 southwest Western Australia strains from the first and the second clusters (strains highlighted by the gray rectangle) among Australasian strains. The node belonging to the non-Australasian strains (indicated by the gray circle, containing 124 strains) has been collapsed. The consistency index for the tree was 0.1620, and the homoplasy index was 0.8380.

We then undertook a phylogenetic analysis of the genomes of the 22 southwest Western Australia isolates with a set of global genomes to determine the phylogeographical origin of the southwest Western Australia isolates. Within our global phylogeny, isolates from the Avon Valley and surrounding area were highly similar and nested within their own well-supported clade within the Australian clade (Fig. 5A). Isolates from Australia (Northern Territory, Queensland, and Western Australia) were interspersed throughout the Australasian clade, with some geographical structure being detected in the lineage, and other Western Australia isolates were distantly related to the isolates from the Avon Valley and strains from the surrounding area (Fig. 5B).

### Limited evidence of recombination among the ST-284 isolates.

To determine the contribution of recombination to the limited diversity noted for the ST-284 isolates, we examined the distribution of SNPs using the Gubbins program. We did not identify recombinogenic SNPs or regions associated with recombination in strains (*n* = 10) from the 2017 cluster. However, we found evidence of recombination in two strains from the first cluster: strains MSHR0162 (isolated from a sheep in 1968; number of recombinogenic SNPs = 35, number of recombinogenic SNPs occurring in recombinogenic blocks = 1, and number of bases associated with the recombinogenic block = 14.4 kb) and MSHR0172 (isolated from a dog in 1985; number of recombinogenic SNPs = 5, number of recombinogenic SNPs occurring in recombinogenic blocks = 1, and number of bases associated with the recombinogenic block = 0.56 kb). The Gubbins program also identified SNPs that arose due to point mutations rather than recombination events, and there was evidence for SNPs occurring outside recombinogenic regions for 18 of the southwest Western Australia strains (range, 1 to 144 SNPs).

### Protein-coding regions unique to isolates from southwest Western Australia.

**FIG 6:**
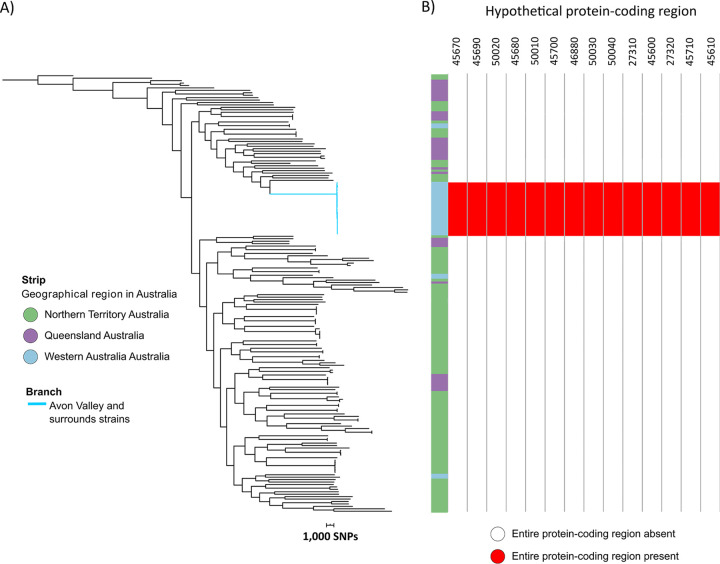
Maximum parsimony phylogenetic analysis of ST-284 isolates with an Australian set of isolates. The phylogeny is based on 206,357 core-genome SNPs. (A) Maximum parsimony phylogeny. The strip delineates the Australian region of origin. (B) Presence/absence of the 14 protein-coding regions that were unique to the ST-284 isolates, as annotated in isolate MSHR0169. Each cell provides the BSR values: 0 represents no alignment (white), and 1 represents identical alignment (red). The consistency index for the tree was 0.1775, and the homoplasy index was 0.8225.

Comparative genomics identified 14 protein-coding regions that were present in all temperate southwest Western Australian isolates but that were absent from a set of tropical Australian B. pseudomallei isolates (Fig. 6). Using the corresponding assemblies annotated with the Prokka program, all 14 protein-coding regions were annotated as hypothetical protein-coding regions and did not have any matches to annotated protein-coding regions in the UniProt database (https://www.uniprot.org/), and so their function is unknown. We did not identify protein-coding region differences between cluster 1 and cluster 2 isolates. We then used Island Viewer (v4; https://www.pathogenomics.sfu.ca/islandviewer/) to determine if the 14 protein-coding regions were located within GIs and demonstrated that the 14 protein-coding regions were located on GIs (Table S4).

### GIs and virulence genes in ST-284 are ubiquitously present or absent.

To further assess the genomic diversity of the 22 ST-284 strains, we examined the distribution of 38 known genomic islands and six variably present virulence genes. Despite the ST-284 isolates being collected over a 51-year time period, all 38 GIs were ubiquitously present or absent in the ST-284 isolate genomes, highlighting the genomic diversity of the ST-284 strains. All 22 isolates contained eight GIs—GI1.1 (comprised of 10 genes; Table S2), GI4.3 (comprised of 15 genes; Table S2), GI7.4 (comprised of 5 genes; Table S2), GI13.4 (comprised of 15 genes; Table S2), GI14 (comprised of 15 genes; Table S2), GI14.1 (comprised of 15 genes; Table S2), GI15e (comprised of 7 genes; Table S2), and GI16b (comprised of 7 genes; Table S2)—and lacked the remaining 30 GIs. At GI1.1, GI7.4, and GI16b, the coding sequences (CDSs) were identical for the ST-284 strains, and minor variations were noted in the CDSs belonging to GI14, GI14.1, GI4.3, and GI15e (see Table S2 for the BLAST score ratio [BSR] scores for the CDSs belonging to the eight identified GIs). Minor variations were present in GI13.4 across the ST-284 strains, and two protein-coding regions belonging to GI13.4 were truncated in MSHR0160 (the sole isolate from Chittering Farm 2): an integrase core domain (protein-coding region BDL_3619; BSR = 0.37) and a putative gp37 (protein-coding region BDL_3620; BSR = 0.29). These truncations were not observed in any other ST-284 strain. Encompassed in the eight GIs (GI1.1, GI4.3, GI7.4, GI13.4, GI14, GI14.1, GI15e, and GI16b) identified in the southwest western Australia strains were genes typically present in GIs, such as genes involved in metabolism, prophage genes, and genes involved in the integration of GIs into the genome, including integrase, transposase, resolvase, and recombinase genes (Table S2). We also determined the presence of the eight GIs in the global strain set (Table S1), and the eight GIs were highly varied across the global data set, with the global genomes either containing complete GIs, lacking GIs, or containing partial GIs.

10.1128/mSystems.00726-20.4 TABLE S2 BSR scores for the eight known GIs identified in the 22 ST-284 strains. Download Table S2, XLSX file, 0.02 MB. Copyright © 2020 Webb et al. 2020 Webb et al. This content is distributed under the terms of the Creative Commons Attribution 4.0 International license.

Lastly, the variably present virulence genes that were screened for in the ST-284 strains were universally present or absent. All 22 of the ST-284 genomes contained *fhaB3*, were positive for the gene for lipopolysaccharide A (LPS A), and contained the B. pseudomallei BimA gene variant (*bimA_Bm_*), further highlighting the similarity of the 22 ST-284 isolates from southwest Western Australia.

## DISCUSSION

While melioidosis is traditionally defined as a disease of the tropics and previous models demonstrated that tropical regions have environments favorable for B. pseudomallei, evidence is mounting for the persistence of B. pseudomallei in both arid regions (20) and temperate regions (10, 21). Since 1966, melioidosis cases have been described in the Avon Valley and surrounding regions (latitude ∼31.6°S). B. pseudomallei was first detected in farm animals, and then in 1980 B. pseudomallei was detected in the environment and in 1992 the first human case was reported (16–19). We hypothesized that additional outbreaks would continue to occur in the region following severe weather events (10). In late January 2017, heavy rain occurred in southwest Western Australia, 17 days prior to the reported alpaca deaths on Moondyne Farm, which fits with the normal incubation period for acute melioidosis of 1 to 21 days and which fits with death usually occurring within several days of illness onset in fatal cases (22). We isolated B. pseudomallei in soil from the farm where the alpacas died, confirming the prolonged presence of B. pseudomallei in the environment of southwest Western Australia. The 10 isolates collected from the 2017 cluster were all ST-284, the same ST identified in the cluster 1 isolates, revealing that the second cluster was not the result of another B. pseudomallei introduction into the region but, rather, represents the long-term low-prevalence endemicity of B. pseudomallei.

Phylogenetic analysis with the B. pseudomallei isolates available revealed the likely origin of the ST-284 melioidosis cases in the four alpacas. The alpaca isolates clustered with culture-positive soil isolates from two separate Moondyne Farm locations, with each alpaca genome being separated from the respective soil samples by only two or four SNPs, suggesting that the alpacas most likely acquired their infections from soil at Moondyne Farm. Necropsy confirmed that the alpacas died from extensive and severe melioidosis pneumonia, consistent with the inhalation of B. pseudomallei which was potentially aerosolized during the severe weather. Studies combining epidemiology (23), climate science (24), clinical and pathological data (25), and bacterial genomics (26, 27) are providing new insights into the postulated shift from percutaneous inoculation to aerosol inhalation following severe weather events. Nevertheless, the proportions of melioidosis cases from inhalation, percutaneous inoculation, and ingestion remain unclear (28).

The clonality observed among the southwest Western Australia strains contrasts to the extensive within-ST diversity observed in settings where melioidosis is highly endemic, such as northern Australia (29). The genetic diversity of the B. pseudomallei genome can arise quickly via recombination within B. pseudomallei and with near-neighbor bacteria. We found evidence of some limited recombination among the ST-284 strains in the first cluster but not among the ST-284 strains in the 2017 cluster. In support of this, no additional STs were identified in the Avon Valley or surrounding environment during the first or second cluster (10), and the lack of genetically diverse strains in the environment potentially hindered genetic diversification through recombination. Another explanation for limited recombination in the southwest Western Australian isolates is that under suboptimal or harsh conditions, the bacteria enter a dormant state and so are mostly not metabolically active and are thus not recombining with neighbors (30). In support of dormancy and the limited recombination, we found no unique protein-coding regions in the isolates of the 2017 cluster when their sequences were compared to those of the isolates in the first cluster, which would have likely arisen via recombination events. We hypothesize that the genetic diversity described here has most likely accumulated by point mutation during replication bursts occurring during short periods of favorable environmental conditions, such as with heavy rainfall. In support of this, our genomic analysis identified point mutations outside of recombinogenic regions in almost all the ST-284 strains.

It remains unknown as to when or how B. pseudomallei was introduced into the Avon Valley region of southwest Western Australia. A separate and unrelated cluster of five cutaneous human melioidosis cases in suburban southwest Australia beginning in 2012 also remains unexplained (21). Our global phylogeny demonstrated that all ST-284 strains clustered on a separate branch within the Australasian clade and that all shared a recent common ancestor. It is clear that ST-284 is of Australian origin and that a single introduction into the Avon Valley and the surrounding region is likely, as previously proposed (10). We were unable to identify a likely origin of the ST-284 strains due to deep Australian evolutionary nodes being unsupported and the lack of a close relative. The true geographical origin of ST-284 cannot be determined until it or closely related STs have been identified elsewhere in the Australian environment outside of southwest Western Australia.

Speculation about the importation of B. pseudomallei into southwest Western Australia via horses has been made (10, 19, 31). In 1965, two horses were imported into southwest Western Australia from tropical Kimberley in northern Western Australia, and autopsy confirmed that the horses died from melioidosis. All clinical isolates from those horses were discarded, so the genotypes of the isolates from that scenario were not available. Further environmental sampling of the Kimberley region may therefore shed light on the origin of ST-284. Alternatively, B. pseudomallei may have been introduced into southwest Western Australia via an extreme weather event. In Australia, B. pseudomallei can become aerosolized during severe weather events, such as cyclones, and severe weather has been implicated in the movement of B. pseudomallei in Australia (32), Taiwan (27), and, more recently, the Caribbean (11). While it is uncertain if weather is directly related to the original arrival of B. pseudomallei to southwest Western Australia, the unusually high level of rainfall in late January 2017 was very likely the driver of the outbreak in the alpacas at Moondyne Farm. In support of this theory, the frequency of melioidosis cases has been shown to fluctuate with rainfall, with increased case rates being associated with periods of higher, more intense rainfall (24). During periods of heavy rainfall and increased surface discharge, B. pseudomallei can be leached out of the soil, which may lead to an increased risk of transmission through contact with broken skin and contaminated soil or via inhalation or ingestion of contaminated water particles. As outlined by the Australian Bureau of Meteorology, as a result of climate change, southwest Western Australia is projected to experience a future decrease in total annual rainfall, with more time in drought and extreme heat waves but with an increase in intense heavy rainfall events. Therefore, despite the predicted generally drier future for southwest Western Australia, future heavy rainfall events may result in further clusters of melioidosis in this particular region of southwest Western Australia, with inhalational melioidosis being a notable concern following severe weather events, such as cyclones (28). In addition, other as yet unknown foci of endemic environmental B. pseudomallei in this and other subtropical locations may become recognized by clusters of melioidosis cases occurring in animals and/or humans. Nevertheless, the specific mechanisms of activation of latent B. pseudomallei bacteria residing in the farm soil, bacterial movement within and across the farm environment under the influence of rainfall and local flooding, and the nature and dynamics of aerosolization all remain unclear.

B. pseudomallei is non-spore forming but is a robust pathogen that can persist and survive for decades in diverse environments in the absence of nutrients (33, 34). We have demonstrated the persistence of B. pseudomallei in the environment of southwest Western Australia over a 51-year time period, with limited evolution occurring. It is hypothesized that the bacterium enters a quiescent nonreplicative or minimally replicative state, enabling survival in the nontropical environment of southwest Western Australia. We identified 14 unique hypothetical protein-coding regions in the strains from this region that were absent in other Australian B. pseudomallei. Whether some of these hypothetical protein-coding regions are implicated in the survival of B. pseudomallei in temperate climatic regions requires further functional protein analyses. Of note is the postulated ability of B. pseudomallei and some other non-spore-forming bacteria to exist in the environment in a dormant nonreplicative state. It was hypothesized that the geographical and temporal genomic phylogeny of Francisella tularensis in Sweden was consistent with the bacteria being in a nearly dormant stage with minimal replication in the environment, with replication bursts occurring to explain regional outbreaks over a period of 10 years (35), and this has also been observed for Yersinia pestis, the plague-causing bacterium (36). Weather may also change bacterial generation and replication cycles, which may be higher with higher ambient temperatures (37). In contrast to the findings from temperate southwest Western Australia, high levels of genetic diversity have been observed over multiple spatial scales in tropical Australia and northeast Thailand (38–40).

Estimating the true evolutionary rate of B. pseudomallei is confounded by the “open genome” and the magnitude of lateral gene transfer among strains. The use of molecular clocks assumes that the substitution rates per site are constant through time and that violation of these conditions can lead to erroneous inferences and incorrect evolutionary estimates. We did not observe a clear trend in our 51-year B. pseudomallei data set, finding limited evidence of a correlation between mutation patterns and time. Isolates from the second melioidosis cluster were most closely related to isolates from 24 years earlier, with one 2017 isolate clustering on another clade with isolates from 51 years earlier. The exact rate of B. pseudomallei evolution in the environment is unknown, although it has been predicted *in silico* to be highly varied at the same 10 loci among distinct strains (41). Similarly, variable substitution rates per site over time have been reported for other bacterial species (42, 43). Due to the lack of a correlation between mutation patterns with time and the associated implications for clock models, we did not infer an evolutionary timescale for the B. pseudomallei 51-year isolate set.

In conclusion, we demonstrated the ongoing presence of B. pseudomallei in the Avon Valley and surrounds of southwest Western Australia, which was unmasked by fatal cases of melioidosis occurring in alpacas and a parrot after unusually high rainfall in January 2017. We used genomics to characterize the genetic changes to the B. pseudomallei isolates from this region of temperate Australia recovered over a period spanning 51 years. All isolates belonged to a single ST and were highly related, with limited evidence of recombination and with conserved GIs. Time and geographical region did not correlate with the mutation patterns, with two isolates from the 1980s being most closely related to the 2017 isolates. These findings challenge the idea that, at least for B. pseudomallei, temporal and geographical distance correlate with evolutionary relatedness. Melioidosis has remained endemic in this temperate region of Australia for over half a century, and further animal and human cases of melioidosis are likely in the future, especially following heavy rainfall events.

## MATERIALS AND METHODS

### Location, epidemiology, and collection of environmental B. pseudomallei isolates.

In 2017, more than 20 alpacas were agisted to Moondyne Farm in southwest Western Australia, coming from two separate nearby properties. In mid-February 2017, the alpacas became unwell and died or were euthanized at Moondyne Farm. This cluster was first noted in the Animal Health Australia surveillance quarterly newsletter (44). Necropsy examination revealed multifocal pulmonary nodules, extensive pulmonary congestion, and hemorrhage in all alpacas examined. Histology examination showed aggregates of small Gram-negative bacillus bacteria present throughout the pulmonary tissue. Lung lesions were cultured from four alpacas (alpacas A to D) (Table 1), and all grew B. pseudomallei, which was also recovered from a vitreous humor sample from alpaca C. During this period, a scarlet macaw parrot residing in Gidgegannup (approximately 50 km south of Moondyne Farm) also died from melioidosis, with B. pseudomallei being cultured from a tissue sample.

On 15 May 2017, the owner of Moondyne Farm collected soil samples (each weighing approximately 200 g and taken from 20 to 30 cm below the soil surface) from 10 locations across Moondyne Farm (Fig. 2; see also Fig. S1 in the supplemental material). The soil samples were shipped to the Menzies School of Health Research melioidosis laboratory in Darwin, Australia, for processing.

10.1128/mSystems.00726-20.1 FIG S1 Locations of the B. pseudomallei-positive samples. (A) Location 7 (the soil sample was taken just above a brackish creek line); (B) location 9; (C) location 10 (the sample was taken from waterlogged soil). Download FIG S1, TIF file, 1.8 MB. Copyright © 2020 Webb et al. 2020 Webb et al. This content is distributed under the terms of the Creative Commons Attribution 4.0 International license.

10.1128/mSystems.00726-20.2 FIG S2 Midpoint-rooted recombination-adjusted ST-284 phylogeny. The scale bar indicates the number of substitutions per site. Branch colors indicate cluster 1 or cluster 2 (with the first cluster occurring between 1966 and 1992 and the second cluster occurring in 2017), the inner strip delineates the location of isolation, the outer strip delineates the isolate source, and the outer strip is followed by the year in which the sample was collected. Download FIG S2, TIF file, 2.5 MB. Copyright © 2020 Webb et al. 2020 Webb et al. This content is distributed under the terms of the Creative Commons Attribution 4.0 International license.

### Bacterial growth, DNA extraction, and B. pseudomallei confirmation.

Culture of B. pseudomallei was performed using previously described methods (26, 45, 46), and B. pseudomallei was confirmed with a real-time PCR assay targeting a B. pseudomallei-specific 115-bp segment within the type 3 secretion system 1 (TTS1) gene (47). Following B. pseudomallei confirmation, genomic DNA was extracted from purified B. pseudomallei colonies (48).

### Sequencing, quality control, and assembly.

For this study, we sequenced 10 new B. pseudomallei isolates. Four of these were environmental isolates collected at Moondyne Farm (from three locations, with two isolates being from a single location), and six were animal isolates (five alpaca isolates, including two from a single alpaca, and one scarlet macaw parrot isolate). The 10 isolates were sequenced on an Illumina NovaSeq 6000 sequencing instrument (Illumina, Inc., San Diego, CA), with each sequencing platform generating 150-bp paired-end reads. Analysis included an additional 12 genomes that we had sequenced from the prior cluster from the Avon Valley and surrounds (1966 to 1992) (10) and 296 genomes that represented the global phylogenetic structure and diversity of B. pseudomallei (Table S1). Metadata (ST and geography) corresponding to the global genomes were used to select the 296 global strains to best represent diversity and geography; the 296 genomes belonged to 201 unique STs and were from 38 geographical regions around the globe. Read quality was conducted using the Trimmomatic (v0.39) tool (49) and the FastQC program (https://www.bioinformatics.babraham.ac.uk/projects/fastqc). Following adapter trimming and quality control, high-quality draft assemblies for all southwest Western Australia genomes (*n* = 22) were generated using the MGAP pipeline (https://github.com/dsarov/MGAP---Microbial-Genome-Assembler-Pipeline), and all assemblies were deemed to be of good quality and so were included in downstream genomic analysis (contigs, <200; *N*_50_, >80,000 bp). Final assemblies were annotated with the Prokka (v1.11) program (50).

### MLST.

The multilocus sequence typing (MLST) profiles for the B. pseudomallei isolates were extracted from the sequencing reads using the BIGSdb tool, which is accessible on the B. pseudomallei MLST website (http://pubmlst.org/bpseudomallei/).

### Variant calling, phylogenetics, and identification of recombination events.

We created two phylogenies; the first phylogeny was created to examine the highest number of identifiable variants among the southwest Western Australia isolates and contained solely the ST-284 isolates (*n* = 22). The second phylogeny contained the southwest Western Australia isolates (*n* = 22) and a global set of B. pseudomallei genomes (*n* = 296) and was constructed to determine if the southwest Western Australia isolates belonged to a single clade and to investigate the presumptive phylogeographical origin of the southwest Western Australia isolates. Both phylogenies were based on orthologous biallelic single-nucleotide polymorphisms (SNPs). For the ST-284 analysis, the genome of isolate MSHR0169 was used as the reference genome (10), and for the global phylogeny, the high-quality genome of Australian B. pseudomallei isolate MSHR1153 was used as the reference for read mapping (*N*_50_, 4,032,226 bp; number of contigs, 2; size, 7,312,903 bp) (51). Orthologous SNPs were identified from the Illumina whole-genome sequencing data using the Genome Analysis Toolkit (GATK; v4.1.0.0) (52), which is wrapped in the SPANDx (v3.2) algorithm (53). The SNP variants identified by GATK were used for phylogenetic reconstruction using maximum parsimony (MP) in the PAUP* (v4.0.b5) program (54). Bootstrapping was carried out, using 1,000 replicates, to establish the robustness of the tree branches. Phylogenetic trees were visualized and manipulated using the Interactive Tree of Life (ITOL; https://itol.embl.de). Recombinogenic SNPs were identified with the Gubbins (Genealogies Unbiased by Recombination in Nucleotide Sequences, v.2.3.1) program, using the default parameters (55).

### Hierarchical Bayesian clustering of the southwest Western Australian isolates.

A Bayesian approach was applied to spatially delineate and infer the genetic structure of the southwest Western Australia B. pseudomallei isolates. Hierarchical clustering of the 22 genomes was done using the RhierBAPS (v1.1.2) program, implemented in R (v3.5.1) software with a tree-independent approach, using the core-genome mapping alignment. This method allows the population to be subdivided into groups with closely related genetic backgrounds. Clustering was performed until convergence to a local optimum using two levels with *k* equal to 20 as the prior upper bound for the number of clusters. Higher levels of clustering were then performed based on the first result using *k* equal to 15 to *k* equal to 8. Marginal likelihood values and level 1 and 2 clustering were the same across all *k* values tested, and this is likely due to the clonality of the southwest Western Australia isolates.

### Pan-genome analysis and identification of unique protein-coding regions.

The pan-genome was calculated for the ST-284 isolates from the Avon Valley and surrounding area (*n* = 22) using the large-scale BLAST score ratio (LS-BSR) pipeline (56) with a 0.9 BSR threshold and the blat alignment option (57). The compare_BSR.py option was used to identify coding sequence (CDS) differences between the first and the second clusters. The conserved and variably conserved coding regions identified in the ST-284 strains were screened against an Australian set of genomes (Table S1), using LS-BSR, to identify protein-coding regions unique to the southwest Western Australia strains, using a BSR threshold of 0.9 for presence.

### Identification of 38 genomic islands and six virulence genes.

To further assess the genetic similarity of the 22 southwest Western Australia strains, we determined the presence of known genomic islands (GIs) and virulence genes. We screened for the presence of 38 known B. pseudomallei GIs from two Australian strains (strains MSHR0668 and MSHR0305) that ranged from 6 kb to 97 kb; CDS coordinates for the 38 GIs have previously been reported (58). The variably present virulence genes that we screened for included LPS A (*wbil* to *apaH* in B. pseudomallei K96243 [GenBank accession number NC_006350]), LPS B (*BUC_3392* to *apaH* in B. pseudomallei 579 [GenBank accession number NZ_ACCE01000003]), LPS B2 (*BURP840_LPSb01* to *BURP840_LPSb21* in B. pseudomallei MSHR840 [GenBank accession number GU574442]), *bimA_Bm_* (*BURPS668_A2118* in B. pseudomallei MSHR668 [GenBank accession number NZ_CP009545]), B. pseudomallei *bimA* (*bimA_Bp_*; *BPSS1492* in B. pseudomallei K96243 [GenBank accession number NC_006351.1]), and *fhaB3* (*BPSS2053* in B. pseudomallei K96243 [GenBank accession number NC_006351.1]). The presence of the 38 GIs and six virulence genes was determined using the LS-BSR pipeline with the ls_bsr.py option and the tblastn program (38 GIs and six virulence genes) (56). We used a BSR threshold of ≥0.6 or ≥0.9 for the presence of CDSs belonging to the GIs or virulence genes, respectively. CDSs with scores below this cutoff were deemed absent or variable. Genome assembly annotations of the ST-284 strains were reviewed at CDSs with various BSR scores (0.2, 0.3, 0.4, 0.5, 0.6, 0.7, 0.8, 0.9) to determine the BSR threshold of ≥0.6 for presence of GIs. For the virulence genes, CDSs had a BSR score of either 0 or >0.9, and so a BSR threshold of ≥0.9 was used for presence of virulence genes.

### Data availability.

Read data for all isolates are available in the Sequence Read Archive (SRA) database (Table S1) under accession number PRJNA615049.

10.1128/mSystems.00726-20.5 TABLE S3 BSR scores for the eight GIs across the set of global B. pseudomallei genomes. Download Table S3, XLSX file, 0.1 MB. Copyright © 2020 Webb et al. 2020 Webb et al. This content is distributed under the terms of the Creative Commons Attribution 4.0 International license.

10.1128/mSystems.00726-20.6 TABLE S4 Locus tags for the 14 protein-coding regions unique to the southwest Western Australia isolates. Download Table S4, XLSX file, 0.01 MB. Copyright © 2020 Webb et al. 2020 Webb et al. This content is distributed under the terms of the Creative Commons Attribution 4.0 International license.

## References

[1] White NJ 2003 Melioidosis. Lancet 361:1715–1722. doi:10.1016/S0140-6736(03)13374-0. 12767750

[2] Currie BJ, Ward L, Cheng AC 2010 The epidemiology and clinical spectrum of melioidosis: 540 cases from the 20 year Darwin prospective study. PLoS Negl Trop Dis 4:e900. doi:10.1371/journal.pntd.0000900. 21152057 PMC2994918

[3] Holden MT, Titball RW, Peacock SJ, Cerdeno-Tarraga AM, Atkins T, Crossman LC, Pitt T, Churcher C, Mungall K, Bentley SD, Sebaihia M, Thomson NR, Bason N, Beacham IR, Brooks K, Brown KA, Brown NF, Challis GL, Cherevach I, Chillingworth T, Cronin A, Crossett B, Davis P, DeShazer D, Feltwell T, Fraser A, Hance Z, Hauser H, Holroyd S, Jagels K, Keith KE, Maddison M, Moule S, Price C, Quail MA, Rabbinowitsch E, Rutherford K, Sanders M, Simmonds M, Songsivilai S, Stevens K, Tumapa S, Vesaratchavest M, Whitehead S, Yeats C, Barrell BG, Oyston PC, Parkhill J 2004 Genomic plasticity of the causative agent of melioidosis, *Burkholderia pseudomallei*. Proc Natl Acad Sci U S A 101:14240–14245. doi:10.1073/pnas.0403302101. 15377794 PMC521101

[4] Cheng AC, Currie BJ 2005 Melioidosis: epidemiology, pathophysiology, and management. Clin Microbiol Rev 18:383–416. doi:10.1128/CMR.18.2.383-416.2005. 15831829 PMC1082802

[5] Dance DA 2015 Editorial commentary: melioidosis in Puerto Rico: the iceberg slowly emerges. Clin Infect Dis 60:251–253. doi:10.1093/cid/ciu768. 25270648 PMC4275060

[6] Chewapreecha C, Holden MT, Vehkala M, Valimaki N, Yang Z, Harris SR, Mather AE, Tuanyok A, De Smet B, Le Hello S, Bizet C, Mayo M, Wuthiekanun V, Limmathurotsakul D, Phetsouvanh R, Spratt BG, Corander J, Keim P, Dougan G, Dance DA, Currie BJ, Parkhill J, Peacock SJ 2017 Global and regional dissemination and evolution of *Burkholderia pseudomallei*. Nat Microbiol 2:16263. doi:10.1038/nmicrobiol.2016.263. 28112723 PMC5300093

[7] Katangwe T, Purcell J, Bar-Zeev N, Denis B, Montgomery J, Alaerts M, Heyderman RS, Dance DA, Kennedy N, Feasey N, Moxon CA 2013 Human melioidosis, Malawi, 2011. Emerg Infect Dis 19:981–984. doi:10.3201/eid1906.120717. 23735189 PMC3713813

[8] Limmathurotsakul D, Golding N, Dance DA, Messina JP, Pigott DM, Moyes CL, Rolim DB, Bertherat E, Day NP, Peacock SJ, Hay SI 2016 Predicted global distribution of *Burkholderia pseudomallei* and burden of melioidosis. Nat Microbiol 1:15008. doi:10.1038/nmicrobiol.2015.8. 27571754

[9] Sarovich DS, Garin B, De Smet B, Kaestli M, Mayo M, Vandamme P, Jacobs J, Lompo P, Tahita MC, Tinto H, Djaomalaza I, Currie BJ, Price EP 2016 Phylogenomic analysis reveals an Asian origin for African *Burkholderia pseudomallei* and further supports melioidosis endemicity in Africa. mSphere 1:e00089-15. doi:10.1128/mSphere.00089-15. PMC4863585 27303718

[10] Chapple SNJ, Sarovich DS, Holden MTG, Peacock SJ, Buller N, Golledge C, Mayo M, Currie BJ, Price EP 2016 Whole-genome sequencing of a quarter-century melioidosis outbreak in temperate Australia uncovers a region of low-prevalence endemicity. Microb Genom 2:e000067. doi:10.1099/mgen.0.000067. 28348862 PMC5343139

[11] Hall CM, Jaramillo S, Jimenez R, Stone NE, Centner H, Busch JD, Bratsch N, Roe CC, Gee JE, Hoffmaster AR, Rivera-Garcia S, Soltero F, Ryff K, Perez-Padilla J, Keim P, Sahl JW, Wagner DM 2019 *Burkholderia pseudomallei*, the causative agent of melioidosis, is rare but ecologically established and widely dispersed in the environment in Puerto Rico. PLoS Negl Trop Dis 13:e0007727. doi:10.1371/journal.pntd.0007727. 31487287 PMC6748447

[12] Wiersinga WJ, Currie BJ, Peacock SJ 2012 Melioidosis. N Engl J Med 367:1035–1044. doi:10.1056/NEJMra1204699. 22970946

[13] Limmathurotsakul D, Wongratanacheewin S, Teerawattanasook N, Wongsuvan G, Chaisuksant S, Chetchotisakd P, Chaowagul W, Day NP, Peacock SJ 2010 Increasing incidence of human melioidosis in northeast Thailand. Am J Trop Med Hyg 82:1113–1117. doi:10.4269/ajtmh.2010.10-0038. 20519609 PMC2877420

[14] Cottew GS 1950 Melioidosis in sheep in Queens land; a description of the causal organism. Aust J Exp Biol Med Sci 28:677–683. doi:10.1038/icb.1950.70. 14838792

[15] Rimington RA 1962 Melioidosis in north Queensland. Med J Aust 49:50–53. doi:10.5694/j.1326-5377.1962.tb76106.x. 14492316

[16] Chapple SN, Price EP, Sarovich DS, McRobb E, Mayo M, Kaestli M, Spratt BG, Currie BJ 2016 Burkholderia pseudomallei genotype distribution in the Northern Territory, Australia. Am J Trop Med Hyg 94:68–72. doi:10.4269/ajtmh.15-0627. 26526925 PMC4710447

[17] Currie B, Smith-Vaughan H, Golledge C, Buller N, Sriprakash KS, Kemp DJ 1994 Pseudomonas pseudomallei isolates collected over 25 years from a non-tropical endemic focus show clonality on the basis of ribotyping. Epidemiol Infect 113:307–312. doi:10.1017/s0950268800051736. 7523158 PMC2271530

[18] Currie B 1993 Medicine in tropical Australia. Med J Aust 158:612–615. 10.5694/j.1326-5377.1993.tb137630.x 8479379

[19] Golledge CL, Chin WS, Tribe AE, Condon RJ, Ashdown LR 1992 A case of human melioidosis originating in south-west Western Australia. Med J Aust 157:332–334. doi:10.5694/j.1326-5377.1992.tb137192.x. 1279367

[20] Yip TW, Hewagama S, Mayo M, Price EP, Sarovich DS, Bastian I, Baird RW, Spratt BG, Currie BJ 2015 Endemic melioidosis in residents of desert region after atypically intense rainfall in central Australia, 2011. Emerg Infect Dis 21:1038–1040. doi:10.3201/eid2106.141908. 25988301 PMC4451904

[21] Merritt AJ, Peck M, Gayle D, Levy A, Ler YH, Raby E, Gibbs TM, Inglis TJ 2016 Cutaneous melioidosis cluster caused by contaminated wound irrigation fluid. Emerg Infect Dis 22:1420–1427. doi:10.3201/eid2208.151149. PMC4982149 27438887

[22] Currie BJ, Fisher DA, Anstey NM, Jacups SP 2000 Melioidosis: acute and chronic disease, relapse and re-activation. Trans R Soc Trop Med Hyg 94:301–304. doi:10.1016/s0035-9203(00)90333-x. 10975006

[23] Currie BJ, Jacups SP 2003 Intensity of rainfall and severity of melioidosis. Emerg Infect Dis 9:1538–1542. doi:10.3201/eid0912.020750. 14720392 PMC3034332

[24] Kaestli M, Grist EPM, Ward L, Hill A, Mayo M, Currie BJ 2016 The association of melioidosis with climatic factors in Darwin, Australia: a 23-year time-series analysis. J Infect 72:687–697. doi:10.1016/j.jinf.2016.02.015. 26945846

[25] Rachlin A, Shilton C, Webb JR, Mayo M, Kaestli M, Kleinecke M, Rigas V, Benedict S, Gurry I, Currie BJ 2019 Melioidosis fatalities in captive slender-tailed meerkats (Suricata suricatta): combining epidemiology, pathology and whole-genome sequencing supports variable mechanisms of transmission with One Health implications. BMC Vet Res 15:458. doi:10.1186/s12917-019-2198-9. 31856823 PMC6921467

[26] Currie BJ, Price EP, Mayo M, Kaestli M, Theobald V, Harrington I, Harrington G, Sarovich DS 2015 Use of whole-genome sequencing to link *Burkholderia pseudomallei* from air sampling to mediastinal melioidosis, Australia. Emerg Infect Dis 21:2052–2054. doi:10.3201/eid2111.141802. 26488732 PMC4622230

[27] Chen PS, Chen YS, Lin HH, Liu PJ, Ni WF, Hsueh PT, Liang SH, Chen C, Chen YL 2015 Airborne transmission of melioidosis to humans from environmental aerosols contaminated with *B. pseudomallei*. PLoS Negl Trop Dis 9:e0003834. doi:10.1371/journal.pntd.0003834. 26061639 PMC4462588

[28] Currie BJ 2015 Melioidosis: evolving concepts in epidemiology, pathogenesis, and treatment. Semin Respir Crit Care Med 36:111–125. doi:10.1055/s-0034-1398389. 25643275

[29] Price EP, Sarovich DS, Smith EJ, MacHunter B, Harrington G, Theobald V, Hall CM, Hornstra HM, McRobb E, Podin Y, Mayo M, Sahl JW, Wagner DM, Keim P, Kaestli M, Currie BJ 2016 Unprecedented melioidosis cases in northern Australia caused by an Asian *Burkholderia pseudomallei* strain identified by using large-scale comparative genomics. Appl Environ Microbiol 82:954–963. doi:10.1128/AEM.03013-15. 26607593 PMC4725268

[30] Lennon JT, Jones SE 2011 Microbial seed banks: the ecological and evolutionary implications of dormancy. Nat Rev Microbiol 9:119–130. doi:10.1038/nrmicro2504. 21233850

[31] Ketterer PJ, Bamford VW 1967 A case of melioidosis in lambs in south Western Australia. Aust Vet J 43:79–80. doi:10.1111/j.1751-0813.1967.tb08886.x.

[32] Inglis TJJ, O'Reilly L, Merritt AJ, Levy A, Heath CH, Heath C 2011 The aftermath of the Western Australian melioidosis outbreak. Am J Trop Med Hyg 84:851–857. doi:10.4269/ajtmh.2011.10-0480. 21633018 PMC3110376

[33] Moore RA, Tuanyok A, Woods DE 2008 Survival of *Burkholderia pseudomallei* in water. BMC Res Notes 1:11. doi:10.1186/1756-0500-1-11. 18710531 PMC2518269

[34] Wuthiekanun V, Smith MD, White NJ 1995 Survival of *Burkholderia pseudomallei* in the absence of nutrients. Trans R Soc Trop Med Hyg 89:491. doi:10.1016/0035-9203(95)90080-2. 8560519

[35] Johansson A, Larkeryd A, Widerstrom M, Mortberg S, Myrtannas K, Ohrman C, Birdsell D, Keim P, Wagner DM, Forsman M, Larsson P 2014 An outbreak of respiratory tularemia caused by diverse clones of *Francisella tularensis*. Clin Infect Dis 59:1546–1553. doi:10.1093/cid/ciu621. 25097081 PMC4650766

[36] Cui Y, Yu C, Yan Y, Li D, Li Y, Jombart T, Weinert LA, Wang Z, Guo Z, Xu L, Zhang Y, Zheng H, Qin N, Xiao X, Wu M, Wang X, Zhou D, Qi Z, Du Z, Wu H, Yang X, Cao H, Wang H, Wang J, Yao S, Rakin A, Li Y, Falush D, Balloux F, Achtman M, Song Y, Wang J, Yang R 2013 Historical variations in mutation rate in an epidemic pathogen, *Yersinia pestis*. Proc Natl Acad Sci U S A 110:577–582. doi:10.1073/pnas.1205750110. 23271803 PMC3545753

[37] Dutta H, Dutta A 2016 The microbial aspect of climate change. Energ Ecol Environ 1:209–232. doi:10.1007/s40974-016-0034-7.

[38] Chantratita N, Wuthiekanun V, Limmathurotsakul D, Vesaratchavest M, Thanwisai A, Amornchai P, Tumapa S, Feil EJ, Day NP, Peacock SJ 2008 Genetic diversity and microevolution of *Burkholderia pseudomallei* in the environment. PLoS Negl Trop Dis 2:e182. doi:10.1371/journal.pntd.0000182. 18299706 PMC2254201

[39] U'Ren JM, Hornstra H, Pearson T, Schupp JM, Leadem B, Georgia S, Sermswan RW, Keim P 2007 Fine-scale genetic diversity among *Burkholderia pseudomallei* soil isolates in northeast Thailand. Appl Environ Microbiol 73:6678–6681. doi:10.1128/AEM.00986-07. 17720819 PMC2075045

[40] Rachlin A, Mayo M, Webb JR, Kleinecke M, Rigas V, Harrington G, Currie BJ, Kaestli M 2020 Whole-genome sequencing of *Burkholderia pseudomallei* from an urban melioidosis hot spot reveals a fine-scale population structure and localised spatial clustering in the environment. Sci Rep 10:5443. doi:10.1038/s41598-020-62300-8. 32214186 PMC7096523

[41] Price EP, Hornstra HM, Limmathurotsakul D, Max TL, Sarovich DS, Vogler AJ, Dale JL, Ginther JL, Leadem B, Colman RE, Foster JT, Tuanyok A, Wagner DM, Peacock SJ, Pearson T, Keim P 2010 Within-host evolution of *Burkholderia pseudomallei* in four cases of acute melioidosis. PLoS Pathog 6:e1000725. doi:10.1371/journal.ppat.1000725. 20090837 PMC2799673

[42] Duchene S, Holt KE, Weill FX, Le Hello S, Hawkey J, Edwards DJ, Fourment M, Holmes EC 2016 Genome-scale rates of evolutionary change in bacteria. Microb Genom 2:e000094. doi:10.1099/mgen.0.000094. 28348834 PMC5320706

[43] Kuo CH, Ochman H 2009 Inferring clocks when lacking rocks: the variable rates of molecular evolution in bacteria. Biol Direct 4:35. doi:10.1186/1745-6150-4-35. 19788732 PMC2760517

[44] Finkelstein J 2017 Western Australia. Anim Health Surv Q 22:44–46. https://animalhealthaustralia.com.au/wp-content/uploads/AHSQ_Q1_2017_FA3_digital.pdf.

[45] Mayo M, Kaesti M, Harrington G, Cheng AC, Ward L, Karp D, Jolly P, Godoy D, Spratt BG, Currie BJ 2011 *Burkholderia pseudomallei* in unchlorinated domestic bore water, tropical northern Australia. Emerg Infect Dis 17:1283–1285. doi:10.3201/eid1707.100614. 21762588 PMC3381386

[46] Limmathurotsakul D, Dance DA, Wuthiekanun V, Kaestli M, Mayo M, Warner J, Wagner DM, Tuanyok A, Wertheim H, Yoke Cheng T, Mukhopadhyay C, Puthucheary S, Day NP, Steinmetz I, Currie BJ, Peacock SJ 2013 Systematic review and consensus guidelines for environmental sampling of *Burkholderia pseudomallei*. PLoS Negl Trop Dis 7:e2105. doi:10.1371/journal.pntd.0002105. 23556010 PMC3605150

[47] Novak RT, Glass MB, Gee JE, Gal D, Mayo MJ, Currie BJ, Wilkins PP 2006 Development and evaluation of a real-time PCR assay targeting the type III secretion system of *Burkholderia pseudomallei*. J Clin Microbiol 44:85–90. doi:10.1128/JCM.44.1.85-90.2006. 16390953 PMC1351940

[48] Currie BJ, Gal D, Mayo M, Ward L, Godoy D, Spratt BG, LiPuma JJ 2007 Using BOX-PCR to exclude a clonal outbreak of melioidosis. BMC Infect Dis 7:68. doi:10.1186/1471-2334-7-68. 17603903 PMC1925088

[49] Bolger AM, Lohse M, Usadel B 2014 Trimmomatic: a flexible trimmer for Illumina sequence data. Bioinformatics 30:2114–2120. doi:10.1093/bioinformatics/btu170. 24695404 PMC4103590

[50] Seemann T 2014 Prokka: rapid prokaryotic genome annotation. Bioinformatics 30:2068–2069. doi:10.1093/bioinformatics/btu153. 24642063

[51] Johnson SL, Baker AL, Chain PS, Currie BJ, Daligault HE, Davenport KW, Davis CB, Inglis TJ, Kaestli M, Koren S, Mayo M, Merritt AJ, Price EP, Sarovich DS, Warner J, Rosovitz MJ 2015 Whole-genome sequences of 80 environmental and clinical isolates of *Burkholderia pseudomallei*. Genome Announc 3:e01282-14. doi:10.1128/genomeA.01282-14. PMC4333647 25676747

[52] McKenna A, Hanna M, Banks E, Sivachenko A, Cibulskis K, Kernytsky A, Garimella K, Altshuler D, Gabriel S, Daly M, DePristo MA 2010 The Genome Analysis Toolkit: a MapReduce framework for analyzing next-generation DNA sequencing data. Genome Res 20:1297–1303. doi:10.1101/gr.107524.110. 20644199 PMC2928508

[53] Sarovich DS, Price EP 2014 SPANDx: a genomics pipeline for comparative analysis of large haploid whole genome re-sequencing datasets. BMC Res Notes 7:618. doi:10.1186/1756-0500-7-618. 25201145 PMC4169827

[54] Swofford DL 2001 PAUP*: phylogenetic analysis using parsimony (*and other methods) 4.0.b5. Sinauer Associates, Sunderland, MA.

[55] Croucher NJ, Page AJ, Connor TR, Delaney AJ, Keane JA, Bentley SD, Parkhill J, Harris SR 2015 Rapid phylogenetic analysis of large samples of recombinant bacterial whole genome sequences using Gubbins. Nucleic Acids Res 43:e15. doi:10.1093/nar/gku1196. 25414349 PMC4330336

[56] Sahl JW, Caporaso JG, Rasko DA, Keim P 2014 The large-scale BLAST score ratio (LS-BSR) pipeline: a method to rapidly compare genetic content between bacterial genomes. PeerJ 2:e332. doi:10.7717/peerj.332. 24749011 PMC3976120

[57] Kent WJ 2002 BLAT—the BLAST-like alignment tool. Genome Res 12:656–664. doi:10.1101/gr.229202. 11932250 PMC187518

[58] Tuanyok A, Leadem BR, Auerbach RK, Beckstrom-Sternberg SM, Beckstrom-Sternberg JS, Mayo M, Wuthiekanun V, Brettin TS, Nierman WC, Peacock SJ, Currie BJ, Wagner DM, Keim P 2008 Genomic islands from five strains of *Burkholderia pseudomallei*. BMC Genomics 9:566. doi:10.1186/1471-2164-9-566. 19038032 PMC2612704

[59] Price EP, Currie BJ, Sarovich DS 2017 Genomic insights into the melioidosis pathogen. Curr Trop Med Rep 4:95–102. doi:10.1007/s40475-017-0111-9.

