# Supplementary figures and images for: A Persisting Nontropical Focus of Burkholderia pseudomallei with Limited Genome Evolution over Five Decades
Source: mSystems. 2020 Nov 10;5(6):e00726-20. doi: 10.1128/mSystems.00726-20 (PMC7657595; doi:10.1128/mSystems.00726-20)

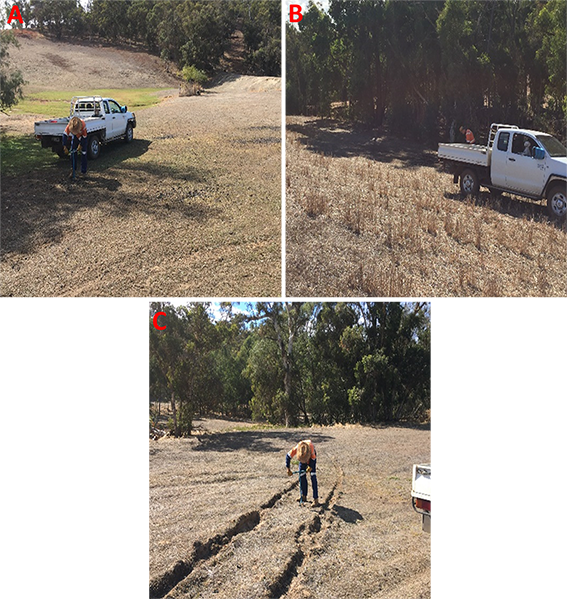

Supplement: FIG S1 [file mSystems.00726-20-sf001.tif]

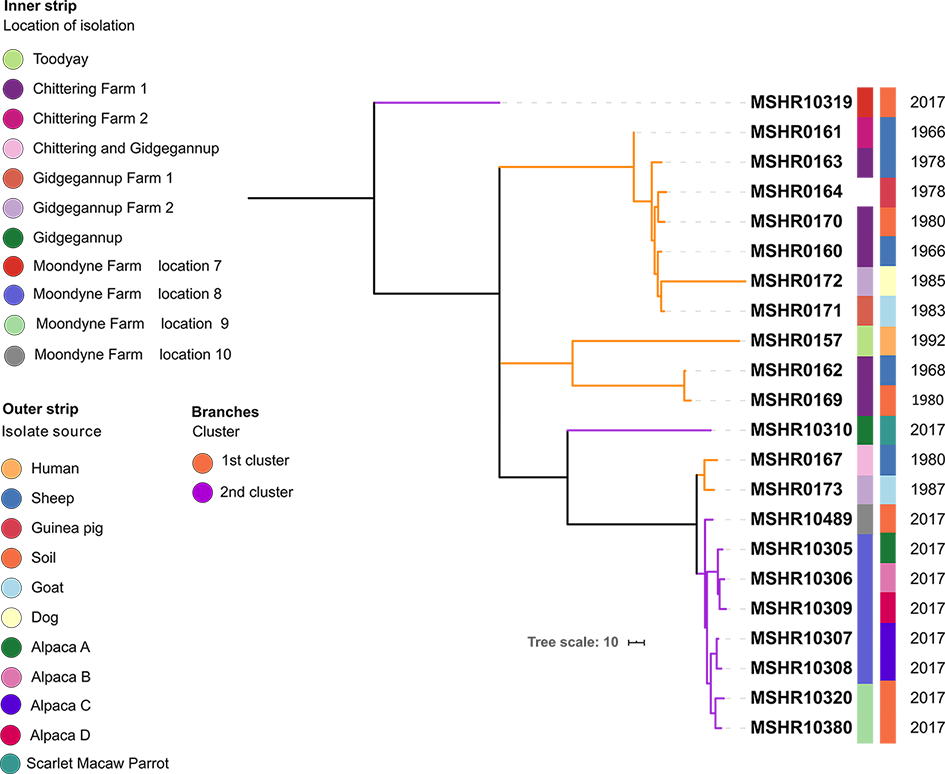

Supplement: FIG S2 [file mSystems.00726-20-sf002.tif]
